# Knowing when to switch off: Promoting psychological well‐being amongst academics working from home

**DOI:** 10.1002/nop2.1201

**Published:** 2022-02-28

**Authors:** Kasim Yaqub Bashir Raja, Sharon Kempson

**Affiliations:** ^1^ 8695 University of Wolverhampton Wolverhampton UK

The academic world has changed significantly due to the coronavirus pandemic. From experience, the pandemic has changed the way into Academics teach, learn and their work dynamics. Multiple lockdowns in various countries are introduced to reduce the spread of the virus caused students' to learn from home and for Academics to teach, mark, learn and research from home (VanLeeuwan et al., 2021). A positive of not travelling to work is the reduction of carbon emissions; however, there have been multiple logistical, educational and procedural changes that Academics have had to adapt to (VanLeeuwan et al., 2021).

Academics have worked hard during the pandemic and are still continuously working hard to ensure that they deliver a high‐quality education and to ensure that they can give an effective level of connectedness online, in a similar fashion to when teaching face to face (VanLeeuwan et al., 2021). Anecdotally, this has involved working long hours, in a space, in their home, to mould curricular material for online teaching, learning and assessment. This has added to the workload of staff during the pandemic, working under very tight deadlines alongside seeing the loss of our friends and family members due to the pandemic (VanLeeuwan et al., 2021).

Furthermore, this has impacted on many Academics ability to shift from working at home to living at home as working from home has potentially desensitized Academics ability to make these shifts, as the need to look at their devices, responding to emails and continuing to work out of hours is occurring, thus creating potential separation anxiety (VanLeeuwan et al., 2021). Anecdotally, this may result in the psychological self‐harm that Academics; due to the need to reduce the amount of workload on them, to continue to work for long hours (Chiew et al., 2018). This, in turn, has a ripple effect where the ability to spend time with family members may have been reduced and to have the time to exercise, watch TV and to escape the work environment may have become more challenging (VanLeeuwan et al., 2021). Despite most of these acts being physical actions, they all play a significant role on the mind, and therefore, reducing the amount of “out‐of‐work” activities may pose their own threats (Chiew et al., 2018).

## 1 CHALLENGES

There are also unique challenges for Academics working from home, which have the potential to cause psychological burden and burnout where switching from homeworking to living at home can add stress, reduce physical activity, cause screen fatigue, eye strain, resulting in a poor diet and reduce social time (Royal Society for Public Health (RSPH), 2021). Additionally, long screen times can affect sleeping patterns that can potentially impact on the mental health of Academics, and placing Academics at risk of morbid diseases (Royal Society for Public Health (RSPH), 2021).

## 2 INTERACTIONS

From experience, there have been multiple barriers to working from home for Academics as opposed to working on campus where immediate access to staff and corridor discussions amongst staff and students enabled an opportunity to allow knowledge sharing and catching up with colleagues. During the period of homeworking, prolongations such as sending meeting requests at mutually agreed dates and times have been difficult due to the busy nature of working from home and the lack of face‐to‐face interactions with the students and staff (VanLeeuwan et al., 2021). Also, asking questions that could take less than a minute to answer face to face, take longer, as it involves writing and sending an email, instead of prompter verbal responses. These add to the additional logistical demands of the role, which could potentially impact Academics negatively and also it can prevent the formation of a community that exists on site (VanLeeuwan et al., 2021).

The balance of teaching face to face with reduced burden on technology, pre‐COVID, significantly changed (VanLeeuwan et al., 2021). Therefore, some Academics who find information technology (IT) systems challenging to work around could have faced many hours working with their institutional IT teams to figure out the functions of particular hardware and software to support teaching, learning, assessment, curriculum design, meetings, emails and administrative duties, which had all been transferred on to technological devices (VanLeeuwan et al., 2021). Anecdotally, this could have led to further stress and it is important to understand from Academics in UK higher education institutions as to how they adapted to this and whether this has influenced some Academics to think about leaving academia or impacted their work‐life balance.

On the contrary, we have identified anecdotally that technology has allowed the increase in networking internationally using technological platforms and using new innovative technological enhanced learning strategies to support student learning. However, from experience, accessing IT systems were not the only challenge, Wi‐Fi issues such as computer incompatibility, signal strength and interference had a detrimental impact on the delivery of online learning. Computer “freeze,” unable to catch all of the conversation due to dropping in and out of the virtual space at crucial moments or at worst unable to connect at all, could potentially cause anxiety and frustration to the Academic (VanLeeuwan et al., 2021). Academics living in rural areas, where average broadband speed is considerately slower than urban areas is still leaving some colleagues at a significant disadvantage when working from home (HM Government, 2019). This could potentially create feelings of loneliness and isolation from the team and students thus potentially reducing the belongingness that staff and students' require.

## 3 SUGGESTIONS

Therefore, it is crucial to have mechanisms in place to avoid the potential psychological harm that could take place from the aforementioned examples, such as: having strict time management to ensure that there are fewer routes to access work emails out of hours. Also, it is beneficial to ensure better diary management to complete work during work hours to prevent working out of hours to allow time for other activities, to enable a work‐life balance (VanLeeuwan et al., 2021). Furthermore, the advantage of having good diary management to enable Academics to complete their day‐to‐day activities in working hours. It is also important to have hours to focus as opposed to being in meetings all day, which could prevent the completion of work in normal working hours (VanLeeuwan et al., 2021). This could lead to continuous overworking and overload, which leads to working overtime (more than 8 hours a day) and thus Academics potentially suffer from the desensitization effect and burnout (Chiew et al., 2018). This could, in turn, affect work‐life balance and impact on performance levels (Chiew et al., 2018).

## 4 AWARENESS AND IMPACT

Further research into this area is required to understand the perspective of Academics and the impact of working from home has had on their life. There is a lack of research focussing on Academics and hence investigating the concepts indicated in the editorial are required. Therefore, this editorial has been written to raise awareness and that we as Academics can be recognized for our hard work and to support Academics who may be suffering negatively. The importance of taking a mixed‐methods approach is crucial to gather quantitative data through completing surveys and then interviewing Academics or having focus groups to expand on some aspects of the survey data identified. The aspects of further investigation include:

Has the pandemic resulted in the increased use of technology amongst some Academics? Has this influenced Academics to consider leaving academia? How have Academics ensured they can adapt to the increased use of technology?

Has working from home affected their performance levels in a positive or negative way? To consider Academics perspectives about the impact on mental health, stress, workload, burnout and way of living as a result of working from home.

Have Academics been working overtime (more than 8 hours a day) whilst working from home? How often has this been the case?

What measures have Academics put into place to know when to switch off and to reduce the risk of potential burnout by working overtime?

This editorial will suggest that research on Academics working from home is crucial and to specifically study on this population because our role as Academics is a multifaceted role that is unique from other roles that also involve working from home. Further clarity is required to address how this has had an impact on the work‐life balance of Academics.

## CONFLICT OF INTEREST

There are no conflicting interest to declare.

## ETHICAL APPROVAL AND PARTICIPANT CONSENT

Ethical approval and participant consent are not required.

## Data Availability

Data sharing is not applicable to this article as no data sets were generated or analysed during the current study.
